# Identification and Validation of a Common Stem Rust Resistance Locus in Two Bi-parental Populations

**DOI:** 10.3389/fpls.2018.01788

**Published:** 2018-11-30

**Authors:** Mandeep S. Randhawa, Ravi P. Singh, Susanne Dreisigacker, Sridhar Bhavani, Julio Huerta-Espino, Matthew N. Rouse, Jayaveeramuthu Nirmala, Maricarmen Sandoval-Sanchez

**Affiliations:** ^1^International Maize and Wheat Improvement Center (CIMMYT), Nairobi, Kenya; ^2^International Maize and Wheat Improvement Center (CIMMYT), Mexico City, Mexico; ^3^Campo Experimental Valle de México INIFAP, Mexico City, Mexico; ^4^Cereal Disease Laboratory, United States Department of Agriculture-Agricultural Research Service, St. Paul, MN, United States; ^5^Colegio de Postgraduados, Texcoco, Mexico

**Keywords:** QTL mapping, SNP, *Pucciniagraminis* f. sp. *tritici*, *Triticumaestivum* L., adult plant resistance

## Abstract

Races belonging to Ug99 lineage of stem rust fungus *Puccinia* *graminis* f. sp. *tritici* (*Pgt*) continue to pose a threat to wheat (*Triticum* *aestivum* L.) production in various African countries. Growing resistant varieties is the most economical and environmentally friendly control measure. Recombinant inbred line (RIL) populations from the crosses of susceptible parent ‘Cacuke’ with the resistant parents ‘Huhwa’ and ‘Yaye’ were phenotyped for resistance at the seedling stage to *Pgt* race TTKSK (Ug99) and in adult plants in field trials at Njoro, Kenya for two seasons in 2016. Using the Affymetrix Axiom breeders SNP array, two stem rust resistance genes, temporarily designated as *SrH* and *SrY*, were identified and mapped on chromosome arm 2BL through selective genotyping and bulked segregant analysis (BSA), respectively. Kompetitive allele specific polymorphism (KASP) markers and simple sequence repeat (SSR) markers were used to saturate chromosome arm 2BL in both RIL populations. *SrH* mapped between markers *cim109* and *cim114* at a distance of 0.9 cM proximal, and *cim117* at 2.9 cM distal. *SrY* was flanked by markers *cim109* and *cim116* at 0.8 cM proximal, and *IWB45932* at 1.9 cM distal. Two Ug99-effective stem rust resistance genes derived from bread wheat, *Sr9h* and *Sr28*, have been reported on chromosome arm 2BL. Infection types and map position in Huhwa and Yaye indicated that *Sr28* was absent in both the parents. However, susceptible reactions produced by resistant lines from both populations against *Sr9h*-virulent race TTKSF+ confirmed the presence of a common resistance locus *Sr9h* in both lines. Test of allelism is required to establish genetic relationships between genes identified in present study and *Sr9h*. Marker *cim117* linked to *SrH* was genotyped on set of wheat lines with Huhwa in the pedigree and is advised to be used for marker assisted selection for this gene, however, a combination of phenotypic and genotypic assays is desirable for both genes especially for selection of *Sr9h* in breeding programs.

## Introduction

Stem rust, caused by *Puccinia graminis* f. sp. *tritici* (*Pgt*), is one of the most damaging fungal diseases of bread and durum wheat (*Triticum aestivum* L. and *Triticum turgidum* ssp. *durum*, (Desf.) Husnot., respectively) (Roelfs et al., 1992). It can cause complete yield losses under severe epidemics if susceptible cultivars are grown in rust hot-spot areas (Saari and Prescott, 1985; Dean et al., 2012). Global wheat production was threatened by stem rust when a highly virulent *Pgt* race known as Ug99 or TTKSK that combined virulence to *Sr31* and various other commonly deployed resistance genes was detected in 1998 in Uganda (Pretorius et al., 2000; Jin et al., 2007, 2008). After its appearance, evaluations of international wheat germplasm and varieties in both field and greenhouse screenings revealed the predominance of wheat susceptibility to race TTKSK (Jin and Singh, 2006; Singh et al., 2006, 2011). In subsequent years, new variants of Ug99 emerged that carry additional virulence to *Sr24* (Jin et al., 2008; Pretorius et al., 2010; Visser et al., 2011), *Sr36* (Jin et al., 2009), and *SrTmp* (Newcomb et al., 2016) placing an even greater number of wheat varieties at risk. Races of the Ug99 race group have already spread over a wide geographical area including 13 countries in the East African highlands, Southern Africa, Yemen, Egypt and Iran, and there is a high chance of further spread into large wheat growing belts of Asia and beyond (Singh et al., 2015). Consequently, the new variants belonging to Ug99 race group and their geographical spread have further reduced the number of effective genes that can be used by breeding programs.

Sixty wheat stem rust resistance genes have a designated gene symbol and a few more carry temporary designations (McIntosh et al., 2016). Five genes, namely *Sr2*, *Sr55* (*Lr67*/*Yr46*/*Pm46*), *Sr56*, *Sr57* (*Lr34*/*Yr18*/*Pm38*), and *Sr58* (*Lr46*/*Yr29*/*Pm39*), confer adult plant resistance (APR) (Singh et al., 2015), and the 34 genes that impart all stage resistance (ASR) to *Pgt* race TTKSK are *Sr9h*, *Sr13a*, *Sr13b*, *Sr15*, *Sr21*, *Sr22*, *Sr24*, *Sr25*, *Sr26*, *Sr27*, *Sr28*, *Sr32*, *Sr33*, *Sr35*, *Sr36*, *Sr37*, *Sr39*, *Sr40*, *Sr42*, *Sr44*, *Sr45*, *Sr46*, *Sr47*, *Sr50*, *Sr51*, *Sr52*, *Sr53*, *Sr59*, *SrCad, SrTA10171*, *SrTA10187*, *SrTA1662*, *SrTmp*, and *Sr1RS^Amigo^* (Jin and Singh, 2006; Jin et al., 2007; Faris et al., 2008; Hiebert et al., 2010; Kolmer et al., 2011; Liu et al., 2011a,b; Qi et al., 2011; Rouse et al., 2011a, 2014; Rouse and Jin, 2011; Ghazvini et al., 2012; Olson et al., 2013a,b; Bansal et al., 2014; Moore et al., 2015; Singh et al., 2015; Rahmatov et al., 2016; Zhang et al., 2017). Interestingly, *Sr8155B1* does not confer resistance to race TTKSK, but does confer resistance to the more recent variants in the Ug99 race group (Nirmala et al., 2017). Only a few resistance genes (*Sr2*, *Sr13a*, *Sr13b*, *Sr25*, *Sr26*, *Sr57*, *Sr58*, *SrCad*, *SrTmp*, *SrND643*, *Sr1RS^Amigo^*, and *Sr8155B1*) were found to confer resistance in adapted germplasm (Jin and Singh, 2006; Singh et al., 2008, 2015; Njau et al., 2010; Hiebert et al., 2011; Basnet et al., 2015; Nirmala et al., 2017; Zhang et al., 2017). The continuous search for new resistance sources and following through with their genetic characterization and strategic deployment is necessary for long-term control of the fast-evolving races of the stem rust fungus.

Bringing widely effective gene combinations into new cultivars is important to avoid the evolution of Ug99 races and minimize potential yield losses. Multiple gene combinations can be achieved more efficiently if DNA markers, tightly linked to target genes, are available. Markers linked to some stem rust resistance genes have been identified which can be useful in marker assisted selection (MAS). Cloning of genes *Sr13a*, *Sr21*, *Sr22, Sr33*, *Sr35*, *Sr45*, *Sr50*, *Sr55*, and *Sr57* has not only enhanced the knowledge of resistance mechanisms but also resulted in the development of diagnostic gene-based markers (Krattinger et al., 2009; Periyannan et al., 2013, 2014; Saintenac et al., 2013; Mago et al., 2015; Moore et al., 2015; Zhang et al., 2017; Chen et al., 2018). Several genes effective against Ug99 were tagged using DNA markers, to name some; *Sr2* (Hayden et al., 2004; Mago et al., 2011), *Sr9h* (Rouse et al., 2014), *SrND643* (Basnet et al., 2015), *Sr22* (Periyannan et al., 2011), *Sr26* (Mago et al., 2005), *Sr25* and *Sr26* (Liu et al., 2010), *Sr28* (Rouse et al., 2012), *Sr35* (Saintenac et al., 2013), *Sr45* (Periyannan et al., 2014), *Sr48* (U. Bansal, University of Sydney, personal communication), *Sr56* (Bansal et al., 2014), *Sr55* (Moore et al., 2015), and *Sr57* (Lagudah et al., 2006). In the last decade, new fast and cost-effective marker technologies emerged as powerful tools, which are very helpful in identifying rust resistance genes and their strategic deployment in breeding.

Observations on ‘Huhwa’ (H), ‘Yaye’ (Y) and their progenies in International Maize and Wheat Improvement Center (CIMMYT) germplasm indicated the presence of ASR to *Pgt* races belonging to the Ug99 group. Furthermore, several high yielding wheat lines derived from Huhwa and Yaye have been distributed in CIMMYT international nurseries. Despite the fact that Huhwa and Yaye carry stem rust resistance genes effective to Ug99 race group, their genetic and molecular characterization remains unclear. Therefore, the present study was undertaken to characterize stem rust resistance in these cultivars using recombinant inbred lines (RIL) populations, and to develop and validate SNP markers closely linked to these genes.

## Materials and Methods

### Plant Material and Development of Mapping Populations

The two RIL populations were developed by crossing stem rust resistant bread wheat lines, Huhwa [CIMMYT germplasm identification number (GID): 5552006] and Yaye (GID: 5343322) each with the susceptible parent Cacuke (GID: 5347441) as the female parent during wheat seasons 2007-08 and 2008-09 at Obregon, Mexico, respectively. The Pedigrees of Huhwa and Yaye are ‘HUW234+*Lr34*/Prinia//Kronstad F2004’ and ‘Yanac/3/Parula/Sara//TSI/Veery#5/4/Croc_1/Ae. squarrosa (224)//Opata’, respectively. The pedigrees of HUW234 and Yanac are ‘HUW12/SPRW//HUW12’ and ‘Jabiru/M-5392-1//M-5392/3/Cook,’ respectively, whereas Cacuke is ‘Canadian/Cunningham//Kennedy.’ Hereafter, Cacuke × Huhwa and Cacuke × Yaye populations are abbreviated as C × H and C × Y, respectively. The F_5_ RIL populations C × H (148 RILs) and C × Y (198 RILs) were developed using single spike descent method modified from the single seed descent method. To develop the population, a single spike from each F_2_ plant, generated from three different F_1_ plants, was harvested under fungicide-protected conditions and advanced to the F_4_ generation by harvesting a single spike in subsequent generations. The F_5_ plots, derived from single F_4_ spikes were then harvested in bulk to obtain sufficient seed of the F_5_ RILs. C × H (3 RILs) and C × Y (27 RILs) did not germinate when populations were planted for seed multiplication at El Batan in 2015. Finally seeds of C × H (145 RILs) and C × Y (171 RILs) were used for all genotypic and phenotypic analyses.

### Greenhouse Evaluations

The parents and RILs of the two populations, C × H and C × Y, were tested against *Pgt* race TTKSK (isolate 04KEN156/04) at the seedling stage in a greenhouse at the United States Department of Agriculture- Agricultural Research Service (USDA-ARS) Cereal Disease Laboratory (CDL), St. Paul, MN, United States following the procedure described in Rouse et al. (2011b). The avirulence/virulence formula of race TTKSK is *Sr24*,*36*,*Tmp*/*Sr5*,*6*,*7b*,*8a*,*9a*,*9b*,*9d*,*9e*,*9g*,*10*,*11*,*17*,*30*,*31*,*38*,*McN* (Jin et al., 2007). Between 20 and 30 seeds of each RIL and the parents were grown for seedling tests. Eight-day old seedlings were inoculated with urediniospores of *Pgt* race TTKSK. Infection types (ITs) on seedlings were recorded 14 days post-inoculation using 0 to 4 scale as described by Stakman et al. (1962). Infection types ‘0,’ ‘1,’ and ‘2’ were considered resistant, whereas ITs ‘3’ to ‘4’ were considered susceptible. RILs were classified in three categories homozygous resistant (HR), homozygous susceptible (HS) and segregating. Two HR and HS RILs from both populations along with respective parents were also tested against *Sr9h*-virulent race TTKSF+ (isolate 09ZIM01-2; Pretorius et al., 2012; Rouse et al., 2014). Tests were repeated twice to avoid any chances of misclassification of RILs in each population.

### Field Evaluations

The two RIL populations and the parents were phenotyped for response to stem rust in field trials at the Njoro research station of Kenya Agricultural and Livestock Research Organization (KALRO) during two wheat growing seasons (2016- off and main seasons). About 5 g seed of each entry was planted in 0.7 m long paired-row flat beds, with 0.3 m spacing between them. Spreaders comprised of a mixture of stem rust susceptible cultivars ‘Cacuke’ and ‘Robin,’ and six-*Sr24* carrying lines (GIDs: 5391050, 5391052, 5391056, 5391057, 5391059, and 5391061) were planted as hill plots on one side of each test plot in the middle of the 0.3 m-wide pathways. Spreaders were also planted along the borders of the experimental field in 1 m plots. Spreaders were inoculated with a field collection of races TTKST and TTKTT belonging to the Ug99 race group (Jin et al., 2008; Singh et al., 2015; Newcomb et al., 2016) by spraying a mixture of urediniospores suspended in water plus Tween 20 suspensions, and needle inoculations with the same suspension as described in Njau et al. (2013). Stem rust responses of each RIL and parents were recorded at post-flowering stage when the susceptible parent displayed moderately susceptible to susceptible (MS to S) responses (Roelfs et al., 1992) with 70% disease severity (DS) following the Modified Cobb’s Scale (Peterson et al., 1948). Responses were recorded a second time about 8 days later.

### Molecular Marker Analysis

Leaf tissues were harvested from young leaves and genomic DNA was extracted using CIMMYT’s laboratory protocols (Dreisigacker et al., 2015). The quality and quantity of DNA was assessed on 1% agarose gels and using a NanoDrop 8000 spectrophotometer (Thermo scientific). Selective genotyping was carried out for parents, 47 resistant and 47 susceptible RILs of C × H population, whereas bulk-segregant analysis (BSA; Michelmore et al., 1991) was performed on pooled DNA of 10 resistant and 10 susceptible RILs along with parents of the C × Y population. Genotyping was conducted using the 35K Affymetrix Axiom Breeders array (Allen et al., 2017) by outsourcing to TraitGenetics GmbH (Germany). The SNPs closely linked to stem rust resistance locus in each population were converted into Kompetitive allele-specific polymorphic (KASP) markers using the automated bioinformatics pipeline, PolyMarker (Ramirez-Gonzalez et al., 2015). KASP markers were then used to verify the targeted SNP polymorphism in the parents and polymorphic markers were applied on the respective mapping population. In addition, 3 KASP markers derived from the SNP markers *IWB45296*, *IWB3891* and *IWB45932* (Wang et al., 2014), linked to *Sr9h* were tested on the respective parents of both populations. Oligos were synthesized in 0.025 μM concentration from Sigma-Aldrich, United States. SNP markers were carried out in a final volume of 8 μl consisting of 3 μl of genomic DNA (30 ng/μl), 4 μl of 2x KASP mix [a mixture of FAM and HEX Fluorescence Resonance Energy Transfer (FRET) cassettes, ROX^TM^ passive dye, an optimized buffer containing MgCl_2_, Taq polymerase and dNTPs], 0.11 μl primer mix containing two allele specific forward primers each with 12 and 30 μM of common primer and 0.89 μl of sterile water. PCR amplification was performed in a GeneAmp PCR System 9700 thermal cycler (Applied Biosystems) using the cycling conditions: 95°C for 15 min for hot-start Taq DNA polymerase activation, followed by a touchdown profile of 9 cycles at 94°C for 20 s and 61°C for 1 min with a 0.6°C reduction per cycle, followed by 38 cycles at 94°C for 20 s and 55°C for 1 min. The reactions were held at 12°C for 3 min. End-point fluorescent images were visualized using the PHERAstar^Plus^ (BMG LABTECH, Germany), and the data were analyzed using KlusterCaller^TM^ software (LGC genomics).

Similarly, 22 simple sequence repeat (SSR) markers namely; *barc101* and *barc159* from Song et al. (2005); *cfd70* and *cfd73* (Sourdille et al., 2001); *gwm16*, *gwm47*, *gwm120*, *gwm382*, *gwm388*, *gwm501* and *gwm526* (Roder et al., 1995, 1998); *wmc51*, *wmc149*, *wmc175*, *wmc317*, *wmc332*, *wmc356*, *wmc361*, *wmc441*, *wmc500*, and *wmc627* (Somers et al., 2004), located on chromosome arm 2BL were also tested on parents, Cacuke, Huhwa and Yaye, to survey polymorphisms between parents and the respective RIL population was genotyped with polymorphic SSRs using respective PCR protocols. In addition, *Sr26*-linked marker *Sr26*#43 (Mago et al., 2005) was tested on ten RILs each of populations C × H and C × Y along with respective parents.

### Data Analyses and Genetic Mapping

Chi-squared analyses were performed to determine the goodness-of-fit of observed segregation with the expected genetic ratios for 1 gene segregation in F_4:5_ RIL population using only the homozygous lines (HR: HS = 1:1). Genetic linkage maps for the stem rust resistance loci were constructed using genotypic data of molecular markers and seedling stem rust data using Inclusive Composite Interval Mapping (ICIM) software (Li et al., 2008). The Kosambi mapping function (Kosambi, 1943) was applied to convert recombination fractions into centimorgans (cM). Markers and respective resistance loci were grouped using a logarithm of odds (LOD) threshold of 10, the linkage maps were ordered and rippled using nearest-neighbor two-opt (nnTwoOpt) and sum of adjacent recombination (SAR) fraction algorithms, respectively.

Associations between markers on chromosome arm 2BL and quantitative resistance were revealed using the DS data recorded during 2016 off and main seasons using the ICIM software. A quantitative trait locus (QTL) was designated to be significant with a LOD value above a threshold calculated through 1000 permutation tests. Linkage map diagrams were prepared and aligned for a visual inspection of map order using MapChart 2.2 software (Voorrips, 2002).

### Validation of Flanking Markers for Marker-Assisted Selection

Closely linked markers to stem rust resistance were used in validation studies by testing on a set of wheat genotypes carrying Huhwa and Yaye in their pedigree, respectively, and the efficacy of the reported markers for marker assisted selection (MAS). Sixty-seven and 44 lines with Huhwa and Yaye in their pedigree respectively, were chosen for validation of flanking markers. These lines were selected from the CIMMYT 9th and 10th Stem Rust Resistance Screening Nursery (SRRSN); the 26th High Rainfall Wheat Screening Nursery (HRWSN); 32nd, 33rd, and 34th Semi-Arid Wheat Screening Nursery (SAWSN); 47th, 48th, and 49th International Bread Wheat Screening Nursery (IBWSN) (Supplementary Tables 1, 2). Seedling IT’s in response to races TTKSK, TTKSF+ and TTKTT (isolate 14KEN58-1, avirulent to *Sr9h*) were assessed at the USDA-ARS Cereal Disease Laboratory (CDL), St. Paul, MN following procedures described in Rouse et al. (2011b). Infection type data were recorded on the 0 to 4 scale of Stakman et al. (1962), where ITs 0, 1, and 2 (or combinations thereof) were considered resistant and ITs 3 and 4 were susceptible. For all wheat lines, stem rust infection response and severity data were also obtained from the international stem rust nurseries evaluated in 2013, 2014, and the 2015 off season in Kenya. The marker haplotype and stem rust phenotype in each line were compared to evaluate the efficiency of the markers in selecting resistance gene.

## Results

### Greenhouse Evaluations

**FIGURE 1:**
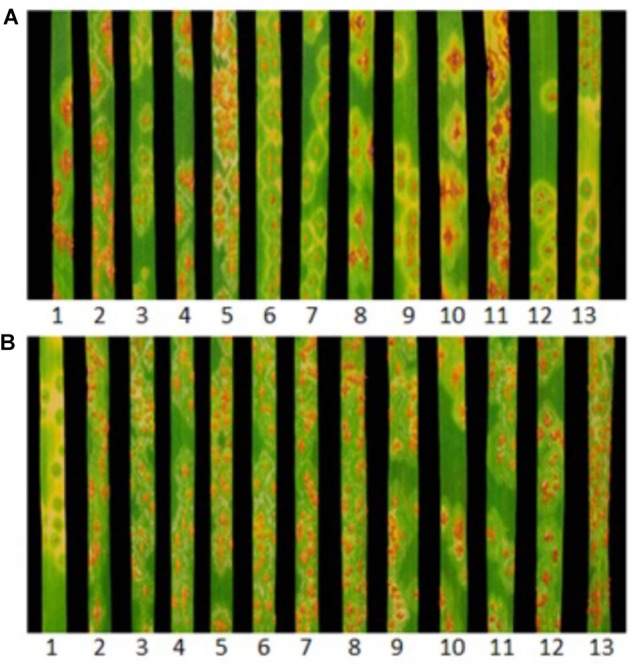
Infection type (IT) responses produced by (1) *Sr31* differential (*Sr31*/6^∗^LMPG), (2) Cacuke, (3) Yaye, (4and 5) Cacuke × Yaye homozygous susceptible (HS) lines, (6 and 7) Cacuke × Yaye homozygous resistant (HR) lines, (8) Cacuke, (9) Huhwa, (10 and 11) Cacuke × Huhwa HS lines, (12 and 13) Cacuke × Huhwa HR lines when tested against *Puccinia graminis* f. sp. *tritici* races **(A)** TTKSK and **(B)** *Sr9h*-virulent TTKSF+.

Huhwa and Yaye displayed resistant ITs 22+ and 2, respectively, in contrast to susceptible IT 3+ for the susceptible parent ‘Cacuke’ when tested at the seedling stage with *Pgt* race TTKSK (Figure 1A). Although both populations were classified into three categories (C × H: 52 HR, 60 HS and 33 segregating;C × Y: 81 HR, 65 HS and 25 segregating) based on seedling ITs, only two categories namely HR and HS, were used for analyses. Monogenic segregation of stem rust resistance was confirmed in C × H [χ^2^_(1:1)_ = 0.58 non-significant and *p* = 0.45] and C × Y [χ^2^_(1:1)_ = 1.76 non-significant and *p* = 0.19] (Table 1). Two HR and HS lines along with respective parents of both populations showed susceptible IT 3+ when tested against *Sr9h*-virulent race TTKSF+ at the seedling stage (Figure 1B). We refer to the single stem rust resistance genes segregating in the C × H and C × Y populations as “*SrH*” and “*SrY*,” respectively.

**Table 1:** Mendelian inheritance of stem rust resistance in Cacuke × Huhwa and Cacuke × Yaye homozygous F_5_ recombinant inbred lines (RILs) when tested with *Puccinia graminis* f. sp. *tritici* (*Pgt*) pathotype TTKSK at seedling stage.

		Number of RILs	
Genotypic classes	Infection type	Observed	Expected	χ^2^_(1:1)_
**Cacuke × Huhwa**				
*SrH SrH*	22+	52	56	0.29
*srH srH*	3+	60	56	0.29
Total		112		0.58^ns^ (*p* = 0.45)
**Cacuke × Yaye**				
*SrY SrY*	2	81	73	0.88
*srY srY*	3+	65	73	0.88
Total		146		1.76^ns^ (*p* = 0.19)


*χ^2^, chi-squared; ns, non-significant.*

### Field Evaluations

**FIGURE 2:**
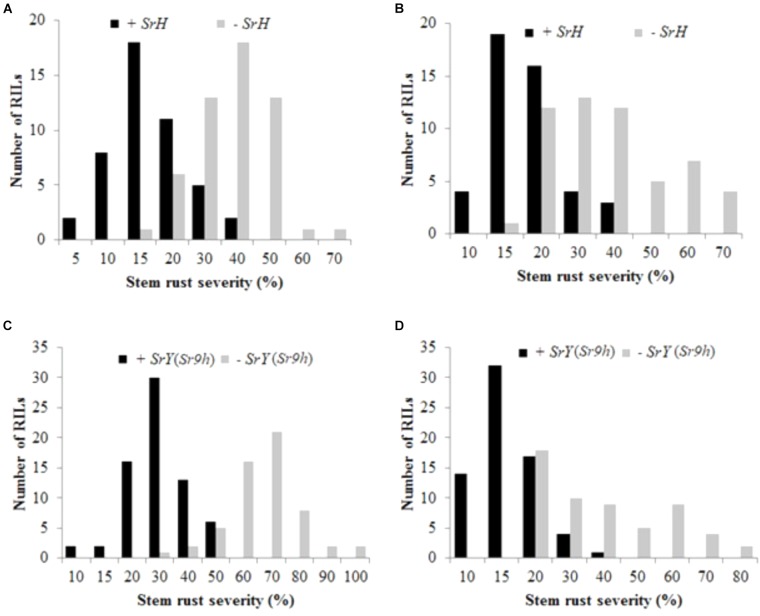
Distribution of stem rust disease severity among recombinant inbred lines of Cacuke × Huhwa with (+) or without (–) *SrH* **(A,B)**; and Cacuke × Yaye with (+) or without (–) *SrY* **(C,D)**. Data were collected during two seasons viz. 2016 off **(A,C)** and main **(B,D)** seasons at Njoro, Kenya.

Uniform disease development was observed in the field nurseries in both seasons in 2016. Terminal disease severity (TDS) and infection response for Cacuke was 70 S, whereas it ranged 10–20 MR for Huhwa and 5–15 RMR for Yaye over two seasons in the adult plant stage. A wide range of infection responses (MR to S) was observed among the RILs in both populations. TDS of RILs in C × H population ranged from 5 to 70% and 10 to 70% during 2016 off and main seasons, respectively (Figures 2A,B). In C × Y population, TDS varied from 10 to 100% and 10 to 80% during 2016 off and main seasons, respectively (Figures 2C,D). Only seedling stem rust data were used to classify lines carrying or not carrying the respective resistance locus in the two populations to avoid any chances of misclassification.

### Mapping of Stem Rust Resistance Locus

#### C × H Population

**FIGURE 3:**
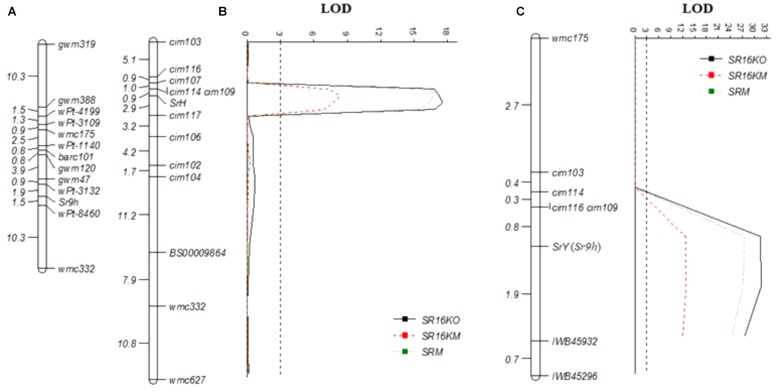
Genetic maps of the *Sr9* region of chromosome 2BL from our data and previously published studies: **(A)** Rouse et al. (2014), **(B)** Cacuke × Huhwa and **(C)** Cacuke × Yaye. Genetic distances between the markers are given in centiMorgan (cM). LOD, Logarithm of odds.

Selective genotyping using 47 each resistant and susceptible RILs along with the parents identified a total of 2,122 polymorphic SNPs distributed across 21 wheat chromosomes. Eighty-three SNPs located on chromosome arm 2BL showed close linkage with stem rust resistance in population C × H using a genetic map of 65.0 cM constructed using ICIM software. Nineteen SNPs linked to *SrH* within 10.0 cM distance were converted into KASP markers designated as *cim* (CIMMYT) namely; *cim101*, *cim102*, *cim103*, *cim104*, *cim105*, *cim106*, *cim107*, *cim108*, *cim109*, *cim110*, *cim111*, *cim112*, *cim113*, *cim114*, *cim115*, *cim116*, *cim117*, *cim118*, and *cim119* (Table 2). In addition, 7 KASP markers (*BS00004405*, *BS00003597*, *BS00009864*, *BS00003589*, *BS00003673*, *BS00010081*, and *BS00009460*) previously mapped on chromosome arm 2BL (Wilkinson et al., 2012), 3 *Sr9h*-linked KASP markers, and 9 KASP markers developed for *SrY* (Table 2) were tested on parents, Cacuke and Huhwa, to verify their polymorphism. A total of 60 markers were screened (the 38 KASP above mentioned and 22 genomic SSRs). Out of them, 19 markers (*cim102*, *cim103*, *cim104*, *cim106*, *cim107*, *cim109*, *cim114*, *cim116*, *cim117*, *cim128*, *BS00009864*, *barc159*, *cfd70*, *gwm47*, *gwm388*, *wmc149*, *wmc317*, *wmc332*, and *wmc627*) showed clear differences between parents and were genotyped on the entire mapping population, C × H. *Sr9h*-linked markers were found to be monomorphic between Cacuke and Huhwa. Using the ICIM mapping software, a partial genetic map of chromosome arm 2BL spanning a distance of 49.8 cM was constructed in which *SrH* was flanked by markers *cim109* and *cim114* at 0.9 cM proximal, and a marker *cim117* at 2.9 cM distal, respectively (Figure 3B). Independent assortment of seven markers (*cim128*, *barc159*, *cfd70, gwm47*, *gwm388*, *wmc149*, and *wmc317*) with respect to *SrH* was observed. The *Sr9h* map from previous study (Rouse et al., 2014) was included for comparison (Figure 3A).

**Table 2:** Primer sequences of KASP markers used to saturate the *SrH*, *SrY*, and *Sr9h* regions on chromosome 2BL.

Gene	Marker	35K SNP id	cM^a^	Allele 1 primer^b^	Allele 2 primer^c^	Common/reverse primer
*SrH*	*cim101*	AX-95652712	103.81	agataacagaacacagtcaaggt	agataacagaacacagtcaaggc	gctggtttgccgttgttgt
	*cim102*	AX-94401499	104.59	attttgtgggcaagccacg	attttgtgggcaagccaca	acatctccaaggtactactaatctc
	*cim103*	AX-94408176	104.59	cagcggcgttgcaaggag	cagcggcgttgcaaggaa	agaaaacatctggaccgaaaatg
	*cim104*	AX-94478727	104.59	tgagatccaggtgcacagg	tgagatccaggtgcacaga	tggtctgtggctgctgaaa
	*cim105*	AX-94585995	104.59	tctgacgttagaagtctcgaatatc	tctgacgttagaagtctcgaatatt	gggaagagacacaggtttcttag
	*cim106*	AX-94641362	104.59	gaaagctcatgcgtgacttc	gaaagctcatgcgtgacttt	ccggcagccacagacagt
	*cim107*	AX-94664270	104.59	gaggaaaaatgccggtgtttcg	gaggaaaaatgccggtgtttcc	gacgcccattaaggagcatgga
	*cim108*	AX-94787485	104.59	tgtcgtgcaagctgctgc	tgtcgtgcaagctgctgg	caccttctggtccatgtcc
	*cim109*	AX-94884026	104.59	gcaaatcaagcgtcccatca	gcaaatcaagcgtcccatcg	gtagcaaaccaagcttatggg
	*cim110*	AX-94897992	104.59	ccgatatttctgaagcatggga	ccgatatttctgaagcatgggg	tgttaagtctcagatacgtgagc
	*cim111*	AX-94934089	104.59	cagcggccttaaagatggcg	cagcggccttaaagatggca	cgaaataaccaaggccaaggaatca
	*cim112*	AX-94939566	104.59	ttgtgctgatggcctatcct	ttgtgctgatggcctatccc	ggcagccacagtttcagtga
	*cim113*	AX-94992638	104.59	tcctccggggtacatgtcc	tcctccggggtacatgtca	tctctgacttgtggagaggaata
	*cim114*	AX-95008732	104.59	acatgcactacatcaccaca	acatgcactacatcaccacg	aggcatctgcatcagctgg
	*cim115*	AX-95102360	104.59	gaacctagctctgtgcccg	gaacctagctctgtgcccc	tgctttgtaactctccacgac
	*cim116*	AX-95234010	104.59	tgttccggatctgtgcacac	tgttccggatctgtgcacat	aacaacggcaaaccagcg
	*cim117*	AX-95629099	104.59	gcggtgtgatcgaagtagtca	gcggtgtgatcgaagtagtcg	tccaggcctccagtggatat
	*cim118*	AX-94399039	NA	cgcaacactcgatctttaattcct	cgcaacactcgatctttaattccc	atggtgttcggccatcctg
	*cim119*	AX-94562871	NA	ccaattatcctctcttactcaaggt	ccaattatcctctcttactcaaggc	acattccgttagagaacagtcact
*SrY*	*cim120*	AX-94433285	72.22	agcttacatatactgcagaatctca	agcttacatatactgcagaatctcg	gttggacgaccatgctcg
	*cim121*	AX-94414057	103.81	tgacatgatgcagacaactcag	tgacatgatgcagacaactcac	gtgccagcaatgaagcatca
	*cim122*	AX-94433997	103.81	cgatgcgcccattccaaac	cgatgcgcccattccaaat	aacctctggaagatggtggc
	*cim123*	AX-94842524	104.59	tcagttcactggcagttctag	tcagttcactggcagttctac	ctgactcatggaagcaatgc
	*cim124*	AX-95107632	153.87	catccttcgtaaacatatgttgtca	catccttcgtaaacatatgttgtcg	aaatgtacaatacttgtccggc
	*cim125*	AX-95242045	153.87	gcggcctcatactgtcaac	gcggcctcatactgtcaat	ccaactttaaagggatctgctac
	*cim126*	AX-95142803	255.63	aatggcatgaggaacagcag	aatggcatgaggaacagcaa	ggtcgaaagcttcatggcg
	*cim127*	AX-94938785	295.13	gcagtcgtccgtccatcg	gcagtcgtccgtccatca	gcagatcgagggcctccc
	*cim128*	AX-94432664	NA	acacggtgcaagaatcaactt	acacggtgcaagaatcaactg	cacagcctcaggtacggata
*Sr9h*	*IWB3891*			cggaacggcgggaggtagta	ggaacggcgggaggtagtg	gctagctaccacatcacggagtaaa
	*IWB45296*			caggagcttggatgaggctacat	aggagcttggatgaggctacac	tggaggctacaacgctatactgcat
	*IWB45932*			aggacgccgaggatcatcagat	ggacgccgaggatcatcagac	ggcgcttccacgggcacaattt


*^a^Consensus genetic map position (Allen et al., 2017); ^*b*^A1 primer labeled with FAM: GAAGGTGACCAAGTTCATGCT; ^*c*^A2 primer labeled with HEX: GAAGGTCGGAGTCAACGGATT, and NA, not available.*

#### C × Y Population

BSA results indicated association of 24 SNPs located on chromosome arm 2BL with stem rust resistance in population C × Y. Nine SNPs were converted into KASP markers *cim120*, *cim121*, *cim122*, *cim123*, *cim124*, *cim125*, *cim126*, *cim127* and *cim128* (Table 2). In total, 38 KASP markers (9 designed for *SrY*, 19 for *SrH*, 3 linked to *Sr9h*, 7 selected from Wilkinson et al. (2012) and 22 SSRs were tested on the parents, Cacuke and Yaye. Finally, 14 markers (*cim103*, *cim109*, *cim114*, *cim116*, *cim128*, *BS00009864*, *IWB3891*, *IWB45296* and *IWB45932*, *barc159*, *cfd70*, *gwm47*, *wmc149*, and *wmc175*) showed clear polymorphism and were genotyped on the entire population Cauke × Yaye. A partial genetic map of chromosome arm 2BL spanned a genetic distance of 6.8 cM with *SrY* flanked by markers *cim109* and *cim116* at 0.8 cM proximal, and marker *IWB45932* at 1.9 cM distal (Figure 3C). Seven markers (*cim128*, *BS00009864*, *IWB3891*, *barc159*, *cfd70*, *gwm47*, and *wmc149*) showed independent assortment with respect to *SrY*. The marker *Sr26*#*43* linked to *Sr26* did not amplify any product in Cacuke, Huhwa, and Yaye.

### Effect of *SrH* and *SrY* on Stem Rust Disease Severity

To study the effect of *SrH* on disease severity, all RILs of the population C × H were reclassified into resistant (+*SrH*) and susceptible (-*SrH*) groups, based on the flanking markers *cim109* and *cim117*. Among 112 RILs, two recombinants were observed between *SrH* and *cim109* whereas 11 recombinants were identified between *SrH* and *cim117*. Based on the flanking markers, 100 non-recombinant RILs, (46 +*SrH* and 54 -*SrH*) were used for this analysis. In all cases, +*SrH* lines displayed MR or M infection responses in the field. Conversely, -*SrH* RILs displayed compatible infection responses (MS, MSS, or S). TDS of +*SrH* was 5 to 40% (mean = 17.2%) and 10 to 40% (mean = 19.3%) in off and main seasons in 2016, respectively, whereas it was 15–70% (mean = 38 and 38.4% during off and main seasons, respectively) in -*SrH* lines during both seasons (Figures 2A,B). The mean differences between the two groups were highly significant (*t* = 11.01, *p* < 0.0001 and *t* = 7.90, *p* < 0.0001 for 2016-off and 2016-main seasons, respectively) indicating that *SrH* confers significant effects on disease severity reduction in addition to conferring a resistant infection response.

Similarly, RILs of C × Y were reclassified into resistant (+*SrY*) and susceptible (-*SrY*) classes, based on the flanking markers *cim109* and *IWB45932*. Among 146 RILs, 18 recombinants were observed between *SrY* and *cim109* whereas 11 between *SrY* and *IWB45932*. Nine recombinants were common between *SrY*, and markers *cim109* and *IWB45932*. Based on flanking markers, 126 non-recombinant RILs, (69 +*SrY* and 57 -*SrY*) were used for this analysis. In all cases, +*SrY* RILs displayed RMR or MR infection responses in the field. In contrast, -*SrY* RILs displayed compatible responses (MS, MSS, or S). The disease severity of +*SrY* was 10–50% (mean = 31%) and 10–40% (mean = 17.2%) in off and main seasons in 2016, respectively, whereas it was 30–100% (mean = 66.8%) and 20–80% (mean = 39.4%) in -*SrY* lines (Figures 2C,D). The mean differences between the two groups were highly significant (*t* = 17.1, *P* < 0.0001 and *t* = 9.13, *P* < 0.0001 for 2016-off and 2016-main seasons, respectively) showing that *SrY* also confers significant effects on disease severity reduction in addition to conferring a resistant infection response.

### Validation of Flanking Markers

Five markers (*cim103*, *cim109*, *cim114*, *cim116*, *cim117*) closely linked to *SrH* were used to genotype 67 CIMMYT advanced breeding lines derived from Huhwa (Supplementary Table 1). Based on the seedling data, 35 and 32 lines were postulated to carry and not-carry *SrH*, respectively. Marker *cim117* amplified *SrH*-linked positive allele A in 32 lines postulated to carry *SrH* whereas 3 lines showed negative allele G. Positive validation of marker *cim117* in 32 lines (out of 35) lines indicated 91.4% efficiency of this marker to identify lines with *SrH*. The seedling ITs of the *SrH* postulated lines ranged from 2- to 2 IT response. Out of 32 lines not postulated to carry *SrH*, 31 lines amplified negative allele G except one line with positive allele A. The other 4 markers were not associated with *SrH* in the advanced breeding lines.

We tested six markers *cim103*, *cim109*, *cim114*, *cim116*, *IWB45932* and *IWB45296* closely linked to *SrY* on a set of 44 wheat lines derived from Yaye (Supplementary Table 2). Marker *IWB45932* amplified *SrY*-linked positive allele T in 14 lines postulated to carry *SrY* whereas one line showed negative allele C. Positive validation of marker *IWB45932* in 14 of the 15 lines showed 93.3% efficiency of this marker to identify lines carrying *SrY*. When tested on 29 lines not postulated to carry *SrY*, marker *IWB45932* amplified *SrY*-linked negative allele C in 13 lines whereas 14 lines carried *SrY*-linked positive allele T. Therefore, IWB45932 alone cannot be used to accurately predict the presence of *SrY*.

## Discussion

In this study, stem rust resistance genes *SrH* and *SrY* were identified in wheat lines Huhwa and Yaye, respectively, and mapped on chromosome arm 2BL using selective genotyping (SG) and bulked segregant analysis (BSA) methods, respectively. Both Huhwa and Yaye have been utilized as common parents in CIMMYT bread wheat improvement program. Therefore, it is expected that *SrH* and *SrY* are already widely present in recently developed wheat lines resistant to *Pgt* races in the Ug99 group.

To this date, five stem rust resistance genes *Sr9*, *Sr16*, *Sr28*, *Sr47*, and *SrWeb* have been mapped on chromosome arm 2BL. There are seven alleles characterized at the *Sr9* locus namely; *Sr9a*, *Sr9b*, *Sr9d*, *Sr9e*, *Sr9f*, *Sr9g*, and *Sr9h*, and each allele demonstrates unique race specificities to *Pgt* races (Green et al., 1960; Knott, 1966; McIntosh and Luig, 1973; Loegering, 1975; Rouse et al., 2014). The gene designation *Sr9c* was originally reserved for a gene that was subsequently designated as *Sr36* on chromosome arm 2BS (McIntosh et al., 1995). *SrWeb* was mapped to chromosome arm 2BL near the *Sr9* locus (Tsilo et al., 2007; Hiebert et al., 2010) and named as *Sr9h* (Rouse et al., 2014).

Six characterized alleles at the *Sr9* locus (*Sr9a*, *Sr9b*, *Sr9d*, *Sr9e*, *Sr9f*, and *Sr9g*) and *Sr16* are not effective against Ug99 race, whereas *Sr9h*, *Sr28*, and *Sr47* confer resistance (Jin et al., 2007; Rouse et al., 2014). Three *Sr9h*-linked KASP markers (*IWB45296*, *IWB3891*, and *IWB45932*) showed no polymorphism between Cacuke and Huhwa. Although SSR marker *gwm47* closely linked to *Sr9h* was polymorphic between Cacuke and Huhwa, the marker could not be integrated into a genetic linkage map with *SrH* due to lack of linkage. No variation shown by *Sr9h*-linked markers between parents and independent assortment of marker *gwm47* with respect to *SrH* suggested that the stem rust resistance locus in Huhwa was unlikely to be *Sr9h*. The *Sr28*-linked marker *wmc332* (Rouse et al., 2012) was mapped more than 30 cM distal to *SrH*. Moreover, IT differences between *Sr28* and *SrH*, indicate potential unlikeness of *SrH* to *Sr28*. *SrH* displayed IT 22+ whereas *Sr28* displayed IT;13- (as reported in Rouse et al., 2014).

In contrast, *Sr9h*-linked markers (*IWB45296* and *IWB45932*) and two SSR markers (*wmc175* and *gwm47*) were found polymorphic between Cacuke and Yaye. Upon genotyping the entire RIL population C × Y, markers *IWB45932* and *IWB45296* were mapped at 1.9 cM proximal and 2.6 cM distal to *SrY*, respectively. Association of *Sr9h*-linked markers with *SrY* indicated possible similarity of *SrY* to *Sr9h*. *Sr28*-linked marker *wmc332* was monomorphic in the parents Cacuke and Yaye, therefore indicating that segregation at the stem rust resistance locus was possibly not associated with *Sr28*. An *Aegilops speltoides* introgression carried *Sr47* which ruled out potential similarity of either *SrY* or *SrH* with *Sr47* (Klindworth et al., 2012) since neither parent carry *Ae. speltoides* in their pedigree. Both *SrH* and *SrY* were mapped on chromosome arm 2BL. There is no common marker between the *SrY* and *SrH* maps that suggests that the position of the genes is different. Both genes are slightly distal to *cim114*. Advanced wheat lines carrying HUW234 and Yanac in their pedigree showed stem rust resistance indicating the most likely source of *SrH* is HUW234 and *SrY* is Yanac. Based upon pedigree information, Yanac could carry stem rust resistance gene *Sr26*, however, marker *Sr26#43* did not amplify any product in resistant parent Yaye, indicating that the gene in Yaye is different from *Sr26*.

Susceptible reactions displayed by seedlings of resistant lines from both populations when tested with *Sr9h* virulent pathotype TTKSF+ confirmed a common resistance locus in both cultivars Huhwa and Yaye which is most likely *Sr9h* identified in wheat cultivar Gabo 56 (Rouse et al., 2014). Gene *Sr9h* is effective against *Pgt* race TTKSK whereas it is ineffective against race TTKSF+. Race TTKSF+, detected in South Africa and Zimbabwe (Pretorius et al., 2012), is virulent on *Sr9h* (Rouse et al., 2014). Similar response shown by Huhwa and Yaye indicated presence of *Sr9h* in both lines.

Association of KASP marker *cim117* on a set of advanced wheat lines postulated to carry *SrH* indicates that this marker could be useful for marker-assisted selection of this gene in breeding. During marker validation process, we concluded that previously known *Sr9h*-linked markers can be used for identification of *Sr9h* or *SrY* together with phenotypic markers as the behavior of *Sr9h* linked markers vary with genetic backgrounds.

RILs carrying *SrH* showed disease severities of 5–40% and 10–40% during 2016 off and main seasons, respectively. However, these lines still displayed incompatible reactions to *Pgt* with infection responses of MR or M. In contrast, in the absence of *SrH*, disease severities of the RILs were 15–70% with corresponding infection responses of MS or S during 2016 off and main seasons. Similar, results were also found for *SrY*-carrying and *SrY* -lacking RILs. As reported by Basnet et al. (2015), disease severity *per se* is not useful for determining the presence or absence of race-specific stem rust resistance genes. Basnet et al. (2015) reported *SrND643*-carrying lines expressing 5–40% disease severities accompanied by R, MR or M infection responses. Similarly, Singh et al. (2011) observed that CIMMYT advanced lines with race-specific genes *Sha7* and *SrTmp* displayed 1–30 and 5–60% disease severities accompanied by R or MR infection responses. Stem rust variation observed in the present study may be due to the presence of quantitative adult plant resistance genes that interact with *SrH* or *SrY*.

## Conclusion

This study identified a common stem rust resistance locus located on chromosome arm 2BL in wheat cultivars Huhwa and Yaye. A robust SNP marker *cim117* closely linked to *SrH* was identified and validated on advanced wheat lines carrying Huhwa in their pedigree. This marker can serve as a breeder friendly tool for marker assisted selection of *SrH* in breeding programs to generate new gene combinations in breeding Ug99 resistant cultivars.

## Kompetitive Allele-Specific Polymorphic (KASP) Markers Data Submission

We attempted to submit KASP markers generated in this study to European Variation Archive (EVA). EVA needs specific requirements including variant quality, filter, information, format etc. for each assay/variant that will only be available if we could have conducted axiom assay in our laboratory. As we outsourced genotyping using 35K axiom breeders’ arrays, this specific information is not available to be included in the submission file to EVA. Markers were generated from publicly available contig sequences developed by International wheat Genome Sequencing Consortium (IWGSC) and available on http://www.cerealsdb.uk.net/cerealgenomics/CerealsDB/axiom_download.php. Markers developed in this study are PCR based markers representing SNPs of contig sequences from 35K axiom breeder’s arrays. Therefore, we decided not to submit these markers to avoid any conflict of interest among wheat scientists.

## Author Contributions

MSR phenotyped the population, did analysis, and wrote the manuscript. MSR, SD, JN, and MS-S genotyped the population with SSR and SNP markers. MSR, RS, SB, and JH-E phenotyped the population at adult plant stage. MNR phenotyped the population at seedling stage.

## Conflict of Interest Statement

The authors declare that the research was conducted in the absence of any commercial or financial relationships that could be construed as a potential conflict of interest.

## Supplementary Material

Table S1 List of advanced wheat lines with Huhwa in their pedigree, their adult plant stem rust response, seedling infection types against *Puccinia graminis* f. sp. *tritici* (*Pgt*) races and haplotypes of *SrH*-linked markers.

Table S2 List of advanced wheat lines with Yaye in their pedigree, their adult plant stem rust response, seedling infection types against *Puccinia graminis* f. sp. *tritici* (*Pgt*) races and haplotypes of *SrY* (*Sr9h*)-linked markers.

## References

[B1] Allen A. M. Winfield M. O. Burridge A. J. Downie R. C. Benbow H. R. Barker G. L. A. (2017). Characterization of a Wheat Breeders’ Array suitable for high-throughput SNP genotyping of global accessions of hexaploid bread wheat (*Triticum aestivum*). *Plant Biotechnol. J.* 15 390–401. 10.1111/pbi.12635 27627182 PMC5316916

[B2] Bansal U. Bariana H. Wong D. Randhawa M. Wicker T. Hayden M. (2014). Molecular mapping of an adult plant stem rust resistance gene *Sr56* in winter wheat cultivar Arina. *Theor. Appl. Genet.* 127 1441–1448. 10.1007/s00122-014-2311-1 24794977

[B3] Basnet B. R. Singh S. Lopez-Vera E. E. Huerta-Espino J. Bhavani S. Jin Y. (2015). Molecular mapping and validation of *SrND643*: a new wheat gene for resistance to the stem rust pathogen Ug99 race group. *Phytopathology* 105 470–476. 10.1094/Phyto-01-14-0016-R 25870921

[B4] Chen S. Zhang W. Bolus S. Rouse M. N. Dubcovsky J. (2018). Identification and characterization of wheat stem rust resistance gene Sr21 effective against the Ug99 race group at high temperature. *PLoS Genet.* 14:e1007287. 10.1371/journal.pgen.1007287 29614079 PMC5882135

[B5] Dean R. Van Kan J. A. Pretorius Z. A. Hammond-Kosack K. E. Di Pietro A. Spanu P. D. (2012). The Top 10 fungal pathogens in molecular plant pathology. *Mol. Plant Pathol.* 13 414–430. 10.1111/j.1364-3703.2011.00783.x 22471698 PMC6638784

[B6] Dreisigacker S. Sehgal D. Luna B. Reyes A. E. Mollins J. (2015). *CIMMYT Wheat Molecular Genetics: Laboratory Protocols and Applications to Wheat Breeding.* Mexico: CIMMYT.

[B7] Faris J. D. Xu S. S. Cai X. Friesen T. L. Jin Y. (2008). Molecular and cytogenetic characterization of a durum wheat-*Aegilops speltoides* chromosome translocation conferring resistance to stem rust. *Chromosome Res.* 16 1097–1105. 10.1007/s10577-008-1261-3 18855109

[B8] Ghazvini H. Hiebert C. W. Zegeye T. Liu S. Dilawari M. Tsilo T. (2012). Inheritance of resistance to Ug99 stem rust in wheat cultivar Norin 40 and genetic mapping of *Sr42*. *Theor. Appl. Genet.* 125 817–824. 10.1007/s00122-012-1874-y 22580967

[B9] Green G. J. Knott D. R. Watson I. A. Pugsley A. T. (1960). Seedling reactions to stem rust of lines of Marquis wheat with substituted genes for rust resistance. *Can. J. Plant. Sci.* 40 524–538. 10.4141/cjps60-069

[B10] Hayden M. J. Kuchel H. Chalmers K. J. (2004). Sequence tagged microsatellites for the *Xgwm533* locus provide new diagnostic markers to select for the presence of stem rust resistance gene *Sr2* in bread wheat (*Triticum aestivum* L.). *Theor. Appl. Genet.* 109 1641–1647. 10.1007/s00122-004-1787-5 15340687

[B11] Hiebert C. W. Fetch T. G. Zegeye T. (2010). Genetics and mapping of stem rust resistance to Ug99 in the wheat cultivar Webster. *Theor. Appl. Genet.* 121 65–69. 10.1007/s00122-010-1291-z 20195568

[B12] Hiebert C. W. Fetch T. G. Zegeye T. Thomas J. B. Somers D. J. Humphreys D. G. (2011). Genetics and mapping of seedling resistance to Ug99 stem rust in Canadian wheat cultivars ‘Peace’ and ‘AC Cadillac’. *Theor. Appl. Genet.* 122 143–149. 10.1007/s00122-010-1430-6 20725713

[B13] Jin Y. Singh R. P. (2006). Resistance in U.S. wheat to recent Eastern African isolates of *Puccinia graminis* f. sp. *tritici* with virulence to resistance gene *Sr31*. *Plant Dis.* 90 476–480. 10.1094/PD-90-0476 30786597

[B14] Jin Y. Singh R. P. Ward R. W. Wanyera R. Kinyua M. Njau P. (2007). Characterization of seedling infection types and adult plant infection responses of monogenic Sr gene lines to race TTKS of *Puccinia graminis* f. sp. tritici. *Plant Dis.* 91 1096–1099. 10.1094/PDIS-91-9-1096 30780647

[B15] Jin Y. Szabo L. J. Pretorius Z. A. Singh R. P. Ward R. Fetch T. (2008). Detection of virulence to resistance gene *Sr24* with race TTKS of *Puccinia graminis* f. sp. *tritici*. *Plant Dis.* 92 923–926. 10.1094/PDIS-92-6-0923 30769714

[B16] Jin Y. Szabo L. J. Rouse M. N. Fetch T. Jr. Pretorus Z. A. Wanyera R. (2009). Detection of virulence to resistance gene *Sr36* within the TTKS race lineage of *Puccinia graminis* f. sp. *tritici*. *Plant Dis.* 93 367–370. 10.1094/PDIS-93-4-0367 30764215

[B17] Klindworth D. L. Niu Z. Chao S. Friesen T. L. Jin Y. Faris J. D. (2012). Introgression and characterization of a goatgrass gene for a high level of resistance to Ug99 stem rust in tetraploid wheat. *G*3 2 665–673. 10.1534/g3.112.002386 22690376 PMC3362296

[B18] Knott D. R. (1966). “The inheritance of stem rust resistance in wheat,” in *Proceedings of the Second International Wheat Genetics Symposium*, ed. MacKey J. (Lund: Heriditas Supplement 2), 156–166.

[B19] Kolmer J. A. Garvin D. F. Jin Y. (2011). Expression of a Thatcher wheat adult plant stem rust resistance QTL on chromosome arm 2BL is enhanced by *Lr34*. *Crop Sci.* 51 526–533. 10.2135/cropsci2010.06.0381

[B20] Kosambi D. D. (1943). The estimation of map distances from recombination values. *Ann. Eugen.* 12 172–175. 10.1111/j.1469-1809.1943.tb02321.x

[B21] Krattinger S. G. Lagudah E. S. Spielmeyer W. Singh R. P. Huerta-Espino J. McFadden H. (2009). A putative ABC transporter confers durable resistance to multiple fungal pathogens in wheat. *Science* 323 1360–1363. 10.1126/science.1166453 19229000

[B22] Lagudah E. S. McFadden H. Singh R. P. Huerta-Espino J. Bariana H. S. Spielmeyer W. (2006). Molecular genetic characterization of the *Lr34*/*Yr18* slow rusting resistance gene region in wheat. *Theor. Appl. Genet.* 114 21–30. 10.1007/s00122-006-0406-z 17008991

[B23] Li H. Ribaut J.-M. Li Z. Wang J. (2008). Inclusive composite interval mapping (ICIM) for digenic epistasis of quantitative traits in biparental populations. *Theor. Appl. Genet.* 116 243–260. 10.1007/s00122-007-0663-5 17985112

[B24] Liu S. Yu L.-X. Singh R. P. Jin Y. Sorrells M. E. Anderson J. A. (2010). Diagnostic and co-dominant PCR markers for wheat stem rust resistance genes *Sr25* and *Sr26*. *Theor. Appl. Genet.* 120 691–697. 10.1007/s00122-009-1186-z 19882111

[B25] Liu W. Jin Y. Rouse M. Friebe B. Gill B. Pumphrey M. O. (2011a). Development and characterization of wheat-*Ae. searsii* Robertsonian translocations and a recombinant chromosome conferring resistance to stem rust. *Theor. Appl. Genet.* 122 1537–1545. 10.1007/s00122-011-1553-4 21347655

[B26] Liu W. Rouse M. Friebe B. Jin Y. Gill B. Pumphrey M. O. (2011b). Discovery and molecular mapping of a new gene conferring resistance to stem rust, *Sr53*, derived from *Aegilops geniculata* and characterization of spontaneous translocation stocks with reduced alien chromatin. *Chromosome Res.* 19 669–682. 10.1007/s10577-011-9226-3 21728140

[B27] Loegering W. Q. (1975). An allele for low reaction to *Puccinia graminis tritici* in Chinese Spring wheat. *Phytopathology* 65:925 10.1094/Phyto-65-925

[B28] Mago R. Bariana H. S. Dundas I. S. Spielmeyer W. Lawrence G. J. Pryor A. J. (2005). Development of PCR markers for the selection of wheat stem rust resistance genes *Sr24* and *Sr26* in diverse wheat germplasm. *Theor. Appl. Genet.* 111 496–504. 10.1007/s00122-005-2039-z 15918008

[B29] Mago R. Brown-Guedira G. Dreisigacker S. Breen J. Jin Y. Singh R. (2011). An accurate DNA marker assay for stem rust resistance gene *Sr2* in wheat. *Theor. Appl. Genet.* 122 735–744. 10.1007/s00122-010-1482-7 21060985

[B30] Mago R. Zhang P. Vautrin S. Simkova H. Bansal U. Luo M. C. (2015). The wheat *Sr50* reveals a rich diversity at a cereal disease resistance locus. *Nat. Plants* 1:15186. 10.1038/nplants.2015.186 27251721

[B31] McIntosh R. A. Dubcovsky J. Rogers J. Morris C. Appels R. Xia X. C. (2016). *Catalogue of Gene Symbols for Wheat: 2015-16 Supplement.* Available at: https://shigen.nig.ac.jp/wheat/komugi/genes/macgene/supplement2015.pdf.

[B32] McIntosh R. A. Luig N. H. (1973). “Recombination between genes for reaction to P. graminis at or near the Sr9 locus,” in *Proceedings of The Fourth International Wheat Genetics Symposium. Agricultural Experiment Station*, eds Sears E. R. Sears L. M. S. (Columbia, MO: University of Missouri), 425–432.

[B33] McIntosh R. A. Wellings C. R. Park R. F. (1995). *Wheat Rusts: an Atlas of Resistance Genes.* East Melbourne: CSIRO Publications, 93–99. 10.1007/978-94-011-0083-0

[B34] Michelmore R. W. Paran I. Kesseli R. V. (1991). Identification of markers linked to disease-resistance genes by bulked segregant analysis: a rapid method to detect markers in specific genomic regions by using segregating populations. *Proc. Natl. Acad. Sci. U.S.A.* 88 9828–9832. 10.1073/pnas.88.21.9828 1682921 PMC52814

[B35] Moore J. W. Herrera-Foessel S. Lan C. Schnippenkoetter W. Ayliffe M. Huerta-Espino J. (2015). A recently evolved hexose transporter variant confers resistance to multiple pathogens in wheat. *Nat. Genet.* 47 1494–1498. 10.1038/ng.3439 26551671

[B36] Newcomb M. Olivera P. D. Rouse M. N. Szabo L. J. Johnson J. Gale S. (2016). Kenyan isolates of *Puccinia graminis* f. sp. tritici from 2008 to 2014: virulence to *SrTmp* in the Ug99 race group and implications for breeding programs. *Phytopathology* 106 729–736. 10.1094/PHYTO-11-14-0302-FI 27019064

[B37] Nirmala J. Saini J. Newcomb M. Olivera P. Gale S. Klindworth D. (2017). Discovery of a novel stem rust resistance allele in durum wheat that exhibits differential reactions to Ug99 isolates. *G*3 7 3481–3490. 10.1534/g3.117.300209 28855282 PMC5633396

[B38] Njau P. N. Bhavani S. Huerta-Espino J. Keller B. Singh R. P. (2013). Identification of QTL associated with durable adult plant resistance to stem rust race Ug99 in wheat cultivar “Pavon 76”. *Euphytica* 190 33–44. 10.1007/s10681-012-0763-4

[B39] Njau P. N. Jin Y. Huerta-Espino J. Keller B. Singh R. P. (2010). Identification and evaluation of sources of resistance to stem rust race Ug99 in wheat. *Plant Dis.* 94 413–419. 10.1094/PDIS-94-4-0413 30754517

[B40] Olson E. L. Rouse M. N. Pumphrey M. O. Bowden R. L. Gill B. S. Poland J. A. (2013a). Introgression of stem rust resistance genes *SrTA10187* and *SrTA10171* from *Aegilops tauschii* to wheat. *Theor. Appl. Genet.* 126 2477–2484. 10.1007/s00122-013-2148-z 23864229

[B41] Olson E. L. Rouse M. N. Pumphrey M. O. Bowden R. L. Gill B. S. Poland J. A. (2013b). Simultaneous transfer, introgression, and genomic localization of genes for resistance to stem rust race TTKSK (Ug99) from *Aegilops tauschii* to wheat. *Theor. Appl. Genet.* 126 1179–1188. 10.1007/s00122-013-2045-5 23377571

[B42] Periyannan K. S. Bansal U. K. Bariana H. S. Pumphrey M. Lagudah E. S. (2011). A robust molecular marker for the detection of shortened introgressed segment carrying the stem rust resistance gene *Sr22* in common wheat. *Theor. Appl. Genet.* 122 1–7. 10.1007/s00122-010-1417-3 20680609

[B43] Periyannan S. Bansal U. Bariana H. Deal K. Luo M. C. Dvorak J. (2014). Identification of a robust molecular marker for the detection of the stem rust resistance gene *Sr45* in common wheat. *Theor. Appl. Genet.* 127 947–955. 10.1007/s00122-014-2270-6 24469473

[B44] Periyannan S. Moore J. Ayliffe M. Bansal U. Wang X. Huang L. (2013). The gene *Sr33*, an ortholog of barley *Mla* genes, encodes resistance to wheat stem rust race Ug99. *Science* 341 786–788. 10.1126/science.1239028 23811228

[B45] Peterson R. F. Campbell A. B. Hannah A. E. (1948). A diagrammatic scale for estimating rust intensity on leaves and stems of cereals. *Can. J. Res.* 26 496–500. 10.1139/cjr48c-033

[B46] Pretorius Z. A. Bender C. M. Visser B. Terefe T. (2010). First report of a *Puccinia graminis* f. sp. *tritici* race virulent to the *Sr24* and *Sr31* wheat stem rust resistance genes in South Africa. *Plant Dis.* 94:784 10.1094/PDIS-94-6-0784C 30754342

[B47] Pretorius Z. A. Singh R. P. Wagoire W. W. Payne T. S. (2000). Detection of virulence to wheat stem rust resistance gene *Sr31* in *Puccinia graminis*. f. sp. *tritici* in Uganda. *Plant Dis.* 84:203. 10.1094/PDIS.2000.84.2.203B 30841334

[B48] Pretorius Z. A. Szabo L. J. Boshoff W. H. P. Herselman L. Visser B. (2012). First report of a new TTKSF race of wheat stem rust (*Puccinia graminis* f. sp. tritici) in South Africa and Zimbabwe. *Plant Dis.* 96:590 10.1094/PDIS-12-11-1027-PDN 30727416

[B49] Qi L. L. Pumphrey M. O. Friebe B. Zhang P. Qian C. Bowden R. L. (2011). A novel Robertsonian translocation event leads to transfer of a stem rust resistance gene (*Sr52*) effective against race Ug99 from *Dasypyrum villosum* into bread wheat. *Theor. Appl. Genet.* 123 159–167. 10.1007/s00122-011-1574-z 21437597

[B50] Rahmatov M. Rouse M. N. Nirmala J. Danilova T. Friebe B. Steffenson B. J. (2016). A new 2DS.2RL Robertsonian translocation transfers stem rust resistance gene *Sr59* into wheat. *Theor. Appl. Genet.* 129 1383–1392. 10.1007/s00122-016-2710-6 27025509

[B51] Ramirez-Gonzalez R. H. Uauy C. Caccamo M. (2015). PolyMarker: a fast polyploid primer design pipeline. *Bioinformatics* 31 2038–2039. 10.1093/bioinformatics/btv069 25649618 PMC4765872

[B52] Roder M. S. Korzun V. Wendehake K. Plaschke J. Tixier M. H. Leroy P. (1998). A microsatellite map of wheat. *Genetics* 149 2007–2023. 9691054 10.1093/genetics/149.4.2007 PMC1460256

[B53] Roder M. S. Plaschke J. Konig S. U. Börner A. Sorrells M. E. Tanksley S. D. (1995). Abundance, variability and chromosomal location of microsatellites in wheat. *Mol. Genet. Genomics* 246 327–333. 10.1007/BF00288605 7854317

[B54] Roelfs A. P. Singh R. P. Saari E. E. (1992). *Rust Diseases of Wheat: Concepts and Methods of Disease Management.* Mexico: CIMMYT.

[B55] Rouse M. N. Jin Y. (2011). Stem rust resistance in A-genome diploid relatives of wheat. *Plant Dis.* 95 941–944. 10.1094/PDIS-04-10-0260 30732109

[B56] Rouse M. N. Nava I. C. Chao S. Anderson J. A. Jin Y. (2012). Identification of markers linked to the race Ug99 effective stem rust resistance gene *Sr28* in wheat (*Triticum aestivum* L.). *Theor. Appl. Genet.* 125 877–885. 10.1007/s00122-012-1879-6 22584633

[B57] Rouse M. N. Nirmala J. Jin Y. Chao S. Fetch T. G. Jr. Pretorius Z. A. (2014). Characterization of Sr9h, a wheat stem rust resistance allele effective to Ug99. *Theor. Appl. Genet.* 127 1681–1688. 10.1007/s00122-014-2330-y 24913360

[B58] Rouse M. N Olson E. Gill B. Pumphrey M. Jin Y. (2011a). Stem rust resistance in *Aegilops tauschii* germplasm. *Crop Sci.* 51 2074–2078. 10.2135/cropsci2010.12.0719 23864229

[B59] Rouse M. N. Wanyera R. Njau P. Jin Y. (2011b). Sources of resistance to stem rust race Ug99 in spring wheat germplasm. *Plant Dis.* 95 762–766. 10.1094/PDIS-12-10-0940 30731910

[B60] Saari E. E. Prescott J. M. (1985). “World distribution in relation to economic losses,” in *The Cereal Rusts: Diseases, Distribution, Epidemiology and Control* Vol. 2 eds Roelfs A. P. Bushnell W. R. (Orlando,FL: Academic Press), 259–298. 10.1016/B978-0-12-148402-6.50017-1

[B61] Saintenac C. Zhang W. Salcedo A. Rouse M. N. Trick H. N. Akhunov E. (2013). Identification of wheat gene Sr35 that confers resistance to Ug99 stem rust race group. *Science* 341 783–786. 10.1126/science.1239022 23811222 PMC4748951

[B62] Singh R. P. Hodson D. P. Huerta-Espino J. Jin Y. Bhavani S. Njau P. (2011). The emergence of Ug99 races of the stem rust fungus is a threat to world wheat production. *Annu. Rev. Phytopathol.* 49 465–481. 10.1146/annurev-phyto-072910-095423 21568701

[B63] Singh R. P. Hodson D. P. Huerta-Espino J. Jin Y. Njau P. Wanyera R. (2008). Will stem rust destroy the world’s wheat crop? *Adv. Agron.* 98 271–309. 10.1016/S0065-2113(08)00205-8

[B64] Singh R. P. Hodson D. P. Jin Y. Huerta-Espino J. Kinyua M. G. Wanyera R. (2006). Current status, likely migration and strategies to mitigate the threat to wheat production from race Ug99 (TTKS) of stem rust pathogen. *CAB Rev. Persp. Agric. Veter. Sci. Nutr. Nat. Resour.* 1 1–13. 10.1079/PAVSNNR20061054

[B65] Singh R. P. Hodson D. P. Jin Y. Lagudah E. S. Ayliffe M. A. Bhavani S. (2015). Emergence and spread of new races of wheat stem rust fungus: continued threat to food security and prospects of genetic control. *Phytopathology* 105 872–884. 10.1094/PHYTO-01-15-0030-FI 26120730

[B66] Somers D. Isaac P. Edwards K. (2004). A high-density microsatellite consensus map for bread wheat (Triticum aestivum L.). *Theor. Appl. Genet.* 109 1105–1114. 10.1007/s00122-004-1740-7 15490101

[B67] Song Q. J. Shi J. R. Singh S. Fickus E. W. Costa J. M. Lewis J. (2005). Development and mapping of microsatellite (SSR) markers in wheat. *Theor. Appl. Genet.* 110 550–560. 10.1007/s00122-004-1871-x 15655666

[B68] Sourdille P. Guyomarc’h H. Baron C. Gandon B. Chiquet V. Artiguenave F. (2001). *Improvement of the Genetic Maps of Wheat using New Microsatellite Markers. Plant & Animal Genome IX, Final Abstracts Guide.* Foster City, CA: Applied Biosystems Press, 167.

[B69] Stakman E. C. Stewart D. M. Loegering W. Q. (1962). *Identification of Physiologic Races of Puccinia graminis var. tritici.* Maryland: United States Department of Agriculture, Agricultural Research Service.

[B70] Tsilo T. J. Jin Y. Anderson J. A. (2007). Microsatellite markers linked to stem rust resistance allele Sr9a in wheat. *Crop Sci.* 47 2013–2020. 10.2135/cropsci2007.02.0087

[B71] Visser B. Herselman L. Park R. F. Karaoglu H. Bender C. M. Pretorius Z. A. (2011). Characterization of two new *Puccinia graminis* f. sp. tritici races within the Ug99 lineage in South Africa. *Euphytica* 179 119–127. 10.1007/s10681-010-0269-x

[B72] Voorrips R. E. (2002). MapChart: software for the graphical presentation of linkage maps and QTLs. *J. Hered.* 93 77–78. 10.1093/jhered/93.1.77 12011185

[B73] Wang S. D. Wong D. Forrest K. Allen A. Chao S. Huang E. (2014). Characterization of polyploid wheat genomic diversity using a high-density 90,000 single nucleotide polymorphism array. *Plant Biotechnol. J.* 12 787–796. 10.1111/pbi.12183 24646323 PMC4265271

[B74] Wilkinson P. A. Winfield M. O. Barker G. L. A. Allen A. M. Burridge A. Coghill J. A. (2012). CerealsDB 2.0: an integrated resource for plant breeders and scientists. *BMC Bioinformatics* 13:219. 10.1186/1471-2105-13-219 22943283 PMC3447715

[B75] Zhang W. Chen S. Abate Z. Nirmala J. Rouse M. N. Dubcovsky J. (2017). Identification and characterization of Sr13, a tetraploid wheat gene that confers resistance to the Ug99 stem rust race group. *Proc. Natl. Acad. Sci. U.S.A.* 114 E9483–E9492. 10.1073/pnas.1706277114 29078294 PMC5692537

